# Cognitive maturity of Portuguese nursing students to intervene in disasters: initial training contribution

**DOI:** 10.1590/1980-220X-REEUSP-2023-0364en

**Published:** 2024-05-20

**Authors:** Paulo Alexandre Figueiredo dos Santos, Rui Carlos Negrão Baptista, Verónica Rita Dias Coutinho, Isabel Cristina Mascarenhas Rabiais

**Affiliations:** 1 Escola Superior de Saúde da Cruz Vermelha Portuguesa, Lisboa, Portugal.; 2 Escola Superior de Enfermagem de Coimbra, Coimbra, Portugal.; 3 Escola Superior de Saúde Atlântica, Lisboa, Portugal.

**Keywords:** Learning, Students, Nursing, Education, Nursing, Teaching, Aprendizaje, Estudiantes de Enfermería, Educación en Enfermería, Enseñanza, Aprendizagem, Estudantes de Enfermagem, Educação em Enfermagem, Ensino

## Abstract

**Objective:**

To understand whether, from the perspective of coordinators/directors of nursing courses and nurses with skills in the field of disasters, nursing students have the necessary cognitive maturity to articulate the various dimensions inherent to the area of disasters, allowing efficient performance.

**Method:**

A study with a qualitative methodological approach, based on inductive reasoning and rigorous phenomenon description, based on exploratory research.

**Results:**

Given the specificity and complexity of these phenomena, the inclusion of the disaster domain in the teaching-learning process, supporting valid knowledge construction and allowing the development and maturity of nursing students’ cognitive processes, is crucial.

**Conclusion:**

Currently, reduced technical-scientific training in the field of disasters in Portugal constitutes a barrier in the development of nursing students’ cognitive maturity, impeding their ability to respond when faced with phenomena of this complexity.

## RESUMO

**Objetivo:**

Compreender se, na perspectiva dos coordenadores/diretores dos cursos de licenciatura em enfermagem e enfermeiros com competências no domínio de desastres, o estudante de enfermagem apresenta a necessária maturidade cognitiva para articular as diversas dimensões inerentes à área de desastres, permitindo um desempenho eficiente.

**Método:**

Estudo de abordagem metodológica qualitativa, sustentado no raciocínio indutivo e na descrição rigorosa dos fenômenos, alicerçado na pesquisa exploratória.

**Resultados:**

Diante da especificidade e da complexidade desses fenômenos, a inclusão do domínio de desastres no processo de ensino-aprendizagem, para subsidiar a construção de conhecimento válido e permitir o desenvolvimento e maturidade dos processos cognitivos do estudante de licenciatura em enfermagem, é determinante.

**Conclusão:**

Atualmente, a redutora formação técnico-científica no domínio de desastres em Portugal constitui uma barreira no desenvolvimento da maturidade cognitiva do estudante de enfermagem, impedindo a sua capacidade de resposta quando confrontado com fenômenos dessa complexidade.

## RESUMEN

**Objetivo:**

Comprender si, desde la perspectiva de los coordinadores/directores de carreras de enfermería y enfermeros con competencias en el área de desastres, el estudiante de enfermería tiene la madurez cognitiva necesaria para articular las diversas dimensiones inherentes al área de desastres, permitiendo desempeño eficiente.

**Método:**

Estudio con enfoque metodológico cualitativo, basado en razonamientos inductivos y descripción rigurosa de los fenómenos, basado en investigación exploratoria.

**Resultados:**

Dada la especificidad y complejidad de estos fenómenos, la inclusión del dominio de desastres en el proceso de enseñanza-aprendizaje, para apoyar la construcción de conocimientos válidos y permitir el desarrollo y madurez de los procesos cognitivos del estudiante de enfermería, es crucial.

**Conclusión:**

Actualmente, la reducida formación técnico-científica en el campo de los desastres en Portugal constituye una barrera en el desarrollo de la madurez cognitiva de los estudiantes de enfermería, impidiendo su capacidad de respuesta ante fenómenos de esta complejidad.

## INTRODUCTION

Disasters are events that are inherently complex and unique, capable of causing damage, economic instability, loss of human life and deterioration of health conditions, in such a way that it exceeds the capacity of a community or society to cope with the situation using its own resources^(1)^.

These are complex situations that lead to ethical dilemmas/conflicts that require understanding multiple perspectives, allowing behavioral changes (less emotional reactivity and greater ability to respect and appreciate different points of view) by: making sometimes difficult decisions in the face of limited resources that impose a difficult balance between the individual and the common good; collaborating with other agents for more informed decision-making; providing, as much as possible, psychological and spiritual support to victims; making life and death decisions; among others^(2)^.

It is understood that intervening in situations of this nature requires healthcare professionals to have knowledge that allows them to be able to manage situations of chaos, uncertainty and anguish, process information (sometimes complex and incomplete) and deal with the abstract, enhancing better decisions. This process requires a dynamic that allows learning, retaining information and responding to the specific challenges of these contexts.

According to available literature, cognitive processes allow memory storage and retrieval, social judgment, decision-making, problem-solving, creativity and flexibility^(3,4,5)^. In the scope of learning, they lead to learning results at the level of knowledge, understanding and development of skills^(2)^.

It appears that developing these capabilities is fundamental as it favors nursing students’ ability to deal with these phenomena competently and face the complexity of these phenomena successfully. Actively contributing to the development of these capabilities enables students to become active and safe beings, revealing greater ease of adaptation^(6)^.

It is important for students to be prepared, with cognitive skills that allow them, in highly complex situations, to use critical thinking in assessment and available information, allowing problem-solving and decision-making at a personal and professional level^(7)^.

Likewise, critical thinking helps purposeful goal-directed thinking, which is intended to formulate judgments based on factual, not conjunctural, evidence. These actions allow students to develop their professional and personal maturity^(8)^. This ability to reflect, attribute meaning to experiences (in the form of thought, feeling or action) and reconsider problem situations as different from what they appear to be proves to be an excellent way of promoting critical thinking and a sense of responsibility^(9)^.

Thus, it is argued that education in disasters is crucial, as it encourages critical thinking (an essential requirement to face contexts characterized by abrupt changes and increasing complexity), mental flexibility (directed by the ability to interpret, analyze, assess, solve problems and assess the results of the thought processes used) and attitudes necessary for elaborating reasoning inherent to the use of these abilities^(10,11)^.

Furthermore, it is important to highlight that, in Portugal, pre-graduate training in nursing is part of the study cycle leading to an academic degree, called a nursing course.

Considering the aforementioned framework, it appears that, in Portugal, promoting initiatives to include this area of knowledge in pedagogical practices in pre-graduate education is incipient^(12)^. One of the determining factors in disaster domain integration ends up being linked to the conviction of coordinators/directors of nursing courses and nurses with knowledge and practical experience in this domain, stating that first-cycle students do not have cognitive maturity (understood as the intellectual capacity to understand and comprehend as well as regulate their emotions in these highly complex contexts), allowing them to respond competently.

In this regard, the present study aims to understand, from the perspective of coordinators/directors of nursing courses and nurses with knowledge and practical experience in the field of disasters, in Portugal, if nursing students have a cognitive maturity that allows them to articulate the different dimensions inherent to the disaster area, respecting their area and competence as well as regulating their emotions in the face of adversity and chaos.

## METHOD

### Study Design

This is a study with a qualitative methodological approach, supported by inductive reasoning and a rigorous phenomenon description, based on exploratory research. The COnsolidated criteria for REporting Qualitative research (COREQ), divided into three domains (research team and reflexivity, research design and analysis and results) and 32 items, was applied, allowing to increase the study rigor and reliability^(13)^.

### Population and Selection Criteria

Intentional sampling was used, and the choice fell on coordinators/directors of nursing courses in public and private schools and nurses with knowledge and practical experience in the field of disasters in Portugal.

With regard to the inclusion criteria for coordinators/directors of nursing courses, they are based on their responsibility, experience and implementation of curriculum objectives, allowing the pursuit and management of the topics to be taught as well as personal characteristics considered fundamental for obtaining rich narratives on the topic under study, allowing relevant reasoning to produce new knowledge.

As for expert nurses, the choice is based on their practical experience in these contexts. All participants were part of national and international organizations with responsibility for prevention and relief actions in the field of disasters, in addition to minimum participation in three nursing course missions and completion in Portugal. These assumptions allow to intuitively expose perspectives capable of generating new knowledge. Experience and being in the context determine a reflection based on evidence and a better awareness and adjustment of the skills that nurses must develop in these contexts^(14)^.

### Data Collection

Semi-structured interviews were used in a face-to-face environment, using the recording technique, applied to coordinators/directors of nursing courses. Hence, a questionnaire was prepared, organized into two thematic chunks: the first consisting of an introductory part with a study general description; a second composed of sociodemographic variables to characterize participants and four open-ended questions. The option to use open-ended questions allowed interviewees to express themselves with complete freedom, using their own vocabulary, providing details and context to the information obtained that enriched the study rigor and depth^(15)^. This process took place between May and September 2018.

These interviews, in addition to clarifying opinions and perspectives related to the phenomenon under study, served as a basis for planning and preparing a second interview guide, structured equally into two thematic chunks (the first, with an introductory part and the second with sociodemographic variables and four open-ended questions), with the aim of being discussed among the group of nurses with knowledge and practical experience in the area of disasters, using the online focus group technique (as some participants were on mission) in November 2018, using Skype^®^ version five, a chat software. Through the use of this technique, in addition to promoting interaction and discussion among the group, promoting contributions to the understanding of the topic under study, it was possible to understand whether the perceptions of coordinators/directors of nursing courses converged or diverged from expert nurses’ perceptions as well as combining uniformity with diversity and, in this way, reaching consensus in relation to the study objective^(16)^. During the focus group’s moderation phase, the researcher had the position of facilitator, allowing the group to maintain the focus on the task of obtaining answers to the questions outlined, whilst seeking not to interfere in the group dynamics^(16)^.

After the conclusion of the first focus group, the information and data collected showed a strong correspondence with information available from other sources, and there was no justification for holding further sessions^(16)^.

### Data Analysis and Treatment

Data from semi-structured interviews and focus group were transcribed and content analyzed, which allowed valid and replicable inferences to be made. Validation of analysis and organization of data obtained from semi-structured interviews and the focus group made it possible to structure the categories (coding systems) and identify recording units (respecting the principles of exhaustiveness and exclusivity, representativeness, homogeneity and productivity). This coding process considered the clipping of texts into units of analysis, the definition of counting rules and classification and aggregation of information into symbolic or thematic categories. It was consolidated through the maintenance, modification or suppression of existing categories, allowing to obtain a system of categories that can be considered, in itself, as the guiding axis of inferred analyses^(16,17)^.

Likewise, in order to maintain the study adequacy and rigor, the following criteria were used, allowing to reduce research inconsistencies and contradictions: credibility (using triangulation of methods as a strategy to increase study solidity and data collection and analysis technique description); transferability (in which a structural organization of the essential phenomenon elements of information collection was outlined in order to facilitate its understanding, as well as the possibility of transferring the results to other contexts with other participants); dependency (process where detailed documentation of all research as well as methodological decisions made was carried out); and confirmability (process that made it possible to assess the extent to which study findings are the product of the investigation focus, through data analysis technique identification and description, in addition to delimiting the context and subjects under study and detailed descriptions of participants, neutralizing any bias by the researcher himself)^(17)^.

### Ethical Aspects

In line with the ethical principles applicable to an investigation of this nature, participants were informed about the study objectives and procedures, safeguarding the right not to participate or to refuse to answer questions that were related to the sphere of privacy. The study obtained a favorable opinion from the *Universidade Católica Portuguesa*, Institute of Health Sciences, Research Ethics Committee, on March 27, 2017 (Opinion 26/2017). All participants signed the informed consent, protecting information secrecy and confidentiality. Coordinators/directors of nursing courses were coded with the letter “I” (for interviewee), followed by the interview number from I1 to I35. The focus group participants, with the letter “F” (for focus), were numbered from F1 to F6, in order to guarantee their anonymity.

## RESULTS

Of the 40 public and private schools, 35 coordinators/directors of nursing courses were included in the sample, as two coordinators/directors did not express interest in participating and three did not respond to the invitation in a timely manner. With regard to expert nurses, six participated.

From the data collected and the floating readings, similarities emerged, which were grouped into two themes and categories (Chart 1):

a) Teaching-learning processes in the field of disasters;
b) General nurse intervention in a disaster situation.

**Chart 1:** Topics and categories – Lisboa, Portugal, 2018.

Topic	Category
**Teaching-learning processes in the field of disasters**	Diagnose to train: training needs of nursing students in the field of disasters; Academia: implications for the teaching-learning process of nursing students in the field of disasters; Interinstitutional and interdisciplinary cooperation: the importance in the pedagogical action of nursing students in the field of disasters; Use of simulated practice in teaching-learning in the disaster area.
**General nurse intervention in a disaster situation**	Regulation of training content in the field of disasters in the nursing discipline; Competency profile of general care nurses for a systematized response in a disaster situation; Reconfiguration of professional culture; Cognitive maturity of nursing course students for a systematized response in the field of disasters.

However, of the eight emerging categories, only the structure of one category related to the initially defined objective is presented.

Based on the highly complex scenario of these phenomena, participants’ narratives reflect the need to include the field of disasters in study plans, in order to favor the development of cognitive skills that allow them to face the demands, constraints and challenges that these contexts require.

(…) *the lack of investment by schools that allows the development of specific knowledge* (of disasters) *in this domain is a current reality. In fact, we schools have not allowed students to develop a path of knowledge construction in this domain that allows for such cognitive maturity, allowing critical thinking, coping and conscious interpretations on this matter* (…) *offering support to assess, make decisions, make judgments. Important dimensions in students’ cognitive development in this domain* (…). (I6)

(…) *the lack of a framework for the disaster area in the degree study plans that allows students to reflect and evaluate these contexts rationally* (…) *on the other hand, in my opinion, I don’t know if undergraduate students have a level of maturity that allows them to mobilize cognitive resources, in order to analyze, assess, argue, solve problems and make decisions in these contexts, i.e., interconnect these different dimensions of these contexts.* (...) *and take a stance in relation to these phenomena of such complexity.* (I8)

(…) *the first cycle student is at a very task-focused stage,* (…) *due to lack of training in this field, making it impossible to develop reasoning and reflective thinking, making it possible to give meaning to this knowledge and, from there, develop attitudes and values to understand these realities, in order to take over, in the future, an active role.* (F1)

(…) *I have doubts that a first cycle student has the cognitive maturity to* (…) *recognize implicit problems and resolve them or formulate a series of alternatives, a process that implies high levels of abstraction.* (F3)

*The integration of this area into the basic training processes of the nursing course can play a fundamental role in this regard. Developing critical thinking, i.e., the ability to critically and reflectively analyze this specific area, allowing objective judgment of response actions to be implemented* (...) *capabilities that require cognitive maturity. Without this work, I find it difficult.* (I33)

## DISCUSSION

From the analysis of participants’ statements, both of coordinators/directors of nursing courses and nurses with knowledge and practical experience in the field of disasters, due to the specificities and complexity of the area, it is clear that it needs to be integrated into students’ teaching-learning processes, allowing developing capabilities, considered essential, for competent intervention in this field. Today, it is argued that if students have a reduced level of cognitive skills, especially when the situations under analysis are complex, they are more prone to intra- and interpersonal conflicts^(18)^. The inability to perceive and feel the conflict, (its) degree of emotionality and reading others’ intentions, so that the response considered most appropriate can be given, can generate behaviors of inaction, indeterminacy or, on the contrary, behaviors of hostility and irritability, which may limit students’ ability, future general nurse, to face change and/or challenge^(18)^.

Thus, it is understood that developing greater literacy in this domain favors students’ resilience, allowing awareness of their intervention, their responsibilities and duties, promoting critical thinking and emotional intelligence and enhancing the adoption of self-protective behaviors^(19)^. In addition to being crucial that the disaster domain can be included in planning educational actions for nursing students, it is equally necessary that the teaching-learning process’s core is based on a logic of developing students’ capabilities until they reach sufficient maturity to manage their interventions in a conscious, structured, balanced and efficient way^(20,21)^.

From this analysis, it is clear that developing emotional intelligence is crucial in this entire process, since emotion perception and regulation allow for greater success in professional performance, specifically in problem-solving. Cognitive and emotional levels are strongly interconnected, allowing students to segregate emotions so that they do not interfere with the performance of their functions^(22)^. This ability can provide students with resources, allowing them to be able to identify and resolve intra, interpersonal and organizational conflicts and create viable alternatives in adverse environments.

Controlling emotions (theirs or those of others) can promote horizons for the ability to negotiate, mediate alternatives and solve conflicts, a process that boosts cognitive growth by allowing recognition of the direct or indirect impact of their actions, i.e., becoming more rational and thinking about the impact of their actions in a critical and autonomous way^(22,23)^. However, the same author states that increasing students’ emotional competence and ability to deal with their own emotions and those of others constitutes a challenge. Students need to cope with diverse experiences and understand that there is no incompatibility between reason and emotion to discover different aspects of their own “self”, their relationship with others and the context that surrounds them. They need to develop the ability to plan intervention methodologies in order to promote psychological, emotional, social, spiritual and cultural development^(23:96)^.

This process additionally requires students’ ability to adapt to difficult situations, knowing how to deal with adversity and retaining the best in each experience. As the ability to adapt to complex situations is not an individual personality trait, nor an immutable construct, it results from students’ ability to mobilize resources in themselves and in the environment that surrounds them to resolve conflicts and/or problem situations. For instance, studies demonstrate that the strategy of reflection on action helps students to reassess their self-esteem, self-control and self-confidence and, thus, look for appropriate ways to face/resolve problem situations, which reveals internal control or available sources of support^(24)^.

At the same time, training in this field enhances responsibility acceptance, which highlights an attitude of greater concentration and a capacity to mobilize resources (internal or external), allowing a better capitalization of thoughts and behaviors to resolve stress-inducing situations or even the ability to tolerate and deal with unforeseen events that tend to persist in achieving their goals^(25)^. This ability contributes positively to motivation, which enables conduct activation, direction and maintenance towards a desired objective, i.e., the feeling of achievement, growth and personal and professional recognition, essential characteristics in disaster contexts, as they enable students to overcome difficult obstacles and achieve objectives with limited resources. It can be seen that the ability to form a decision has a strong relationship with the ability to analyze or rationally consider certain information, forming an opinion and acting accordingly, skills closely related to the critical thinking process.

However, enabling students to understand this type of phenomenon, in all its dimensions, requires experiential learning, i.e., the mere expository transmission of knowledge in this domain, often focusing on abstract and highly complex concepts, proves to be insufficient. It requires methodologies that can reproduce this type of phenomena, ensuring fluent and continuous learning, without compromising student safety. In this regard, it is argued that simulated practice as a teaching-learning methodology is important in this process, since it allows students to develop formal reasoning operations (through stimuli captured by the senses), autonomy, creativity, rationality, intuition (even if acquired through simulation), tolerance to ambiguity and change^(25)^. In addition to increasing knowledge, it allows developing the individual thought process and intellectual development, increasing the ability to transfer knowledge to new situations and, consequently, self-confidence. Furthermore, there are improvements at the level of the communication process, team dynamics, clinical reasoning and decision-making, leadership and safety precautions, capabilities considered fundamental to intervene in the field of disasters efficiently and effectively^(19,20)^.

At the same time, any information or organizational process for complex and ambiguous situations requires communication, enabling vital information transmission for prevention and response processes to situations of this nature. But it also allows to generate trust, credibility, the ability to motivate, express emotions as well as one of the main bases of leadership skills. Leadership is vitally important in performance and motivation, whether at the individual level or at the healthcare team level, favoring flexibility, unity, systemic vision and integrative development. However, it provides inspiration, encouragement and intellectual development, allowing for more effective responses to any sudden and intense change^(26,27,28)^. In the field of disasters, it is crucial that students, future general nurses, are able to adapt to continuous changes with speed, agility and persistence. This process promotes motivation and creativity, but also the ability to plan, organize and manage complex and ambiguous situations (in the face of certain ethical dilemmas), favoring the development of “self” and performance resources, which promotes cognitive maturity^(28)^.

It is therefore understood, from participants’ perspective, that training in the field of disasters must be understood as the “engine” for developing nursing students’ potential/cognitive skills, as it enables the understanding of these phenomena and their dimensions as well as their professional responsibility in these contexts. This process allows them to develop various capabilities, such as stabilized social behavior, allowing better interactions and social transmissions, emotional robustness and resilience in the face of uncertainty and ambiguity, allowing formal operation consolidation.

However, it appears that Nursing Schools’ contribution in Portugal in integrating the field of disasters into training processes remains small, preventing students from developing the necessary cognitive processes, considered crucial for effective performance^(12)^.

As a limitation of this study, we highlighted the importance of expanding the sample of participants in the group of nurses, circumstance that was not possible to achieve due to the contacts made being on mission as well as the lack of adequate or available technological means that would allow them to be part of the online focus group panel.

## CONCLUSION

We consider that the training horizon in the field of disasters, in nursing course’s training processes, is considered decisive in the development of students’ cognitive maturity, due to the enhancing role they have in all other capabilities. Capabilities such as behavioral regulation and emotional self-regulation, in addition to awareness, allowing structured planning of functional strategies and achievement of objectives, and which responsibilities directly enhance motivation and can favor a better understanding of response processes, require a learning process that makes students a “critical thinker”. This exchange of capabilities and resources encourages the emergence of new knowledge, enabling awareness of the specificities of these contexts and contributing to greater cognitive maturity, which favors more effective and efficient performance as well as greater visibility and recognition of the profession in this domain.
